# Optic disc dose reduction in ocular brachytherapy using ^125^I notched COMS plaques: A simulation study based on current clinical practice

**DOI:** 10.1002/acm2.12966

**Published:** 2020-07-12

**Authors:** Yongsook C. Lee, Shih‐Chi Lin, Yongbok Kim

**Affiliations:** ^1^ Department of Radiation Oncology The University of Arizona Tucson AZ USA

**Keywords:** ^125^I, Collaborative Ocular Melanoma Study (COMS), notched plaques, optic disc dose reduction, ocular brachytherapy

## Abstract

**Purpose:**

Although notched Collaborative Ocular Melanoma Study (COMS) plaques have been widely used, optic disc dose reduction by notched COMS plaques has not been discussed in the literature. Therefore, this study investigated optic disc dose reduction in ocular brachytherapy using ^125^I notched COMS plaques in comparison with optic disc dose for ^125^I standard COMS plaques.

**Methods:**

For this simulation study, an in‐house brachytherapy dose calculation program was developed using MATLAB software by incorporating the American Association of Physicists in Medicine Task Group‐43 Update (AAPM TG‐43U1) dosimetry formalism with a line source approximation in a homogeneous water medium and COMS seed coordinates in the AAPM TG 129. Using this program, optic disc doses for standard COMS plaques (from 12 to 22 mm in diameter in 2 mm increments) and notched COMS plaques with one seed removed (Case #1, from 12 to 22 mm) and with two seeds removed (Case #2, from 14 to 22 mm) were calculated as a function of tumor margin‐to‐optic disc distance (DT) for various tumor basal dimensions (BDs) for prescription depths from 1 to 10 mm in 1 mm intervals. A dose of 85 Gy for an irradiation time of 168 h was prescribed to each prescription depth. Then absolute and relative optic disc dose reduction by notched COMS plaques (Cases #1 and #2) was calculated for all prescription depths.

**Results:**

Optic disc dose reduction by notched COMS plaques (Cases #1 and #2) had five unique trends related to maximum optic disc dose reduction and corresponding optimal DT for each BD in each plaque. It increased with increasing prescription depth.

**Conclusions:**

The results presented in this study would enable the clinician to choose an adequate plaque type among standard and notched ^125^I COMS plaques and a prescription depth to minimize optic disc dose.

## 1 INTRODUCTION

A variety of treatment techniques for juxtapapillary choroidal melanoma (a tumor within 1 mm of the optic disc)^1^, ^2^ have been used and such techniques include enucleation, plaque radiotherapy, charged particle (proton or other heavy ions) radiotherapy, stereotactic radiotherapy, and transpupillary thermotherapy.^2^, ^3^ Plaque radiotherapy is a preferred modality to enucleation for medium‐sized tumors (apical height from 2.5 to 10 mm and maximum tumor basal dimension of ≤16 mm) as it allows for eye and vision retention with equivalent tumor control.^4^, ^5^ The Collaborative Ocular Melanoma Study (COMS) randomized trial evaluated ^125^I plaque radiotherapy versus enucleation for medium‐sized choroidal melanomas but excluded juxtapapillary melanoma from the plaque radiotherapy arm and offered enucleation instead.^3^, ^6^ Nevertheless, major institutions have treated juxtapapillary choroidal melanoma using plaque brachytherapy.^1^, ^2^, ^7^

One of the major contraindications for plaque brachytherapy is visual change/loss, and its substantial risk factors were found to be proximity of the tumor to the optic disc and radiation dose to the optic disc.^3^, ^8^, ^9^ To spare the optic disc and consequently, to reduce the contraindication, notched plaques were devised primarily for juxtapapillary or circumpapillary (overhanging the optic disc) tumors.^3^, ^10^ Notched COMS plaques, which have usually one radionuclide seed removed from standard COMS plaques, have been widely used.^11^ Customized notched plaques or slotted plaques were also designed and have been used to accommodate the optic disc better than notched COMS plaques.^3^, ^10^, ^12^ In the literature, visual outcomes for these notched or slotted plaques were reported.^3^, ^10^, ^12^ To the best of our knowledge, however, detailed dosimetry information on radiation dose reduction to the optic disc by the use of notched plaques has not been published before. The purpose of this study, therefore, was to investigate absolute and relative dose reduction to the optic disc in ocular brachytherapy using ^125^I notched COMS plaques in comparison with optic disc dose for ^125^I standard COMS plaques. This investigation was made based on simulations using our in‐house brachytherapy dose calculation program developed in MATLAB software.

Two American Association of Physicists in Medicine (AAPM) Task Group (TG) reports (TG 129^4^ and TG 221^13^) providing guidelines of dose calculation methods in ocular plaque brachytherapy recommend a dual approach which is the AAPM TG‐43 calculation in parallel with a heterogeneous calculation or estimate. Nonetheless, as the recently published AAPM TG‐221 report^13^ explicitly states, *there are currently no treatment planning systems for heterogeneity‐corrected dose calculations for ocular brachytherapy* and current clinical practice for ocular plaque brachytherapy is to use AAPM TG‐43 calculations. In addition, there are no published heterogeneous calculations (i.e., heterogeneity correction factors) for commercially available seed models.^4^, ^13^ Thus, this study was performed based on the current clinical practice (i.e., dose calculations using the AAPM TG‐43 formalism without heterogeneity corrections).

## 2 MATERIALS AND METHODS

### 2.A Configurations of notched COMS plaques

**Fig. 1:**
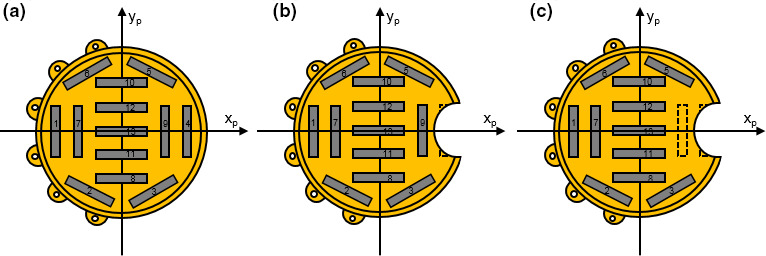
Seed diagrams for (a) 14 mm standard Collaborative Ocular Melanoma Study (COMS) plaque, (b) 14 mm notched COMS plaque with one seed (seed #4) removed and (c) 14 mm notched COMS plaque with two seeds (seeds #4 and #9) removed. The dotted rectangle(s) in (b) and (c) represent removed seed(s).

Seven different‐sized (from 10 to 22 mm in diameter in 2 mm increments) standard COMS plaques^4^ and corresponding notched COMS plaques are currently available. The notched COMS plaques were designed to remove one seed^4^ but two seeds can be removed to reduce optic disc dose further. As shown in the AAPM TG‐129 report, the seed configuration and coordinates of the 10 mm plaque do not allow for seed removal.^4^ Also, the seed configuration of the 12 mm plaque does not allow for two seeds removal.^4^ Hence, six (from 12 to 22 mm) notched plaques for one seed removal and five (from 14 to 22 mm) notched plaques for two seeds removal are options to reduce optic disc dose. Along the X_p_ axis, one side (+X_p_ direction) of notched COMS plaques has a notch for optic disc accommodation and the other side (−X_p_ direction) has suture lugs for a suture on the surface of the eye [Figs. 1(b)–1(c)]. Seed(s) in the notch side are removed to reduce optic disc dose. Table 1 shows seed position number(s) removed from standard COMS plaques in Fig. 1 of the AAPM TG‐129 report.^4^ As an example, seed diagrams for the 14 mm standard COMS plaque and notched COMS plaques with one seed removed and with two seeds removed are shown in Figs. 1(a)–1(c) in order. For one seed removal, seed #4 was removed instead of seed #1. Likewise, for two seeds removal, seed #4 and #9 were removed instead of seeds #1 and #7.

**Table 1:** Seed position number(s) removed from standard Collaborative Ocular Melanoma Study (COMS) plaques. Detailed information on seed positions and configurations for each COMS plaque is found in Fig. 1 of the AAPM TG‐129 report.^4^

Plaque size in diameter (mm)	12	14	16	18	20	22
Case #1 (one seed removal)	3	4	4	5	5	5
Case #2 (two seeds removal)	Unavailable	4, 9	4, 10	5, 12	5, 13	5, 12

### 2.B Eye anatomy

**Fig. 2:**
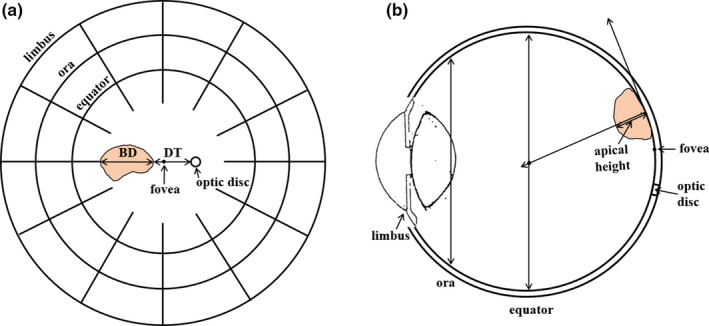
(a) Fundus diagram showing basal dimension and distance from optic disc to the tumor and (b) cross‐section view of the eye showing apical height of the tumor.

Based on current clinical practice for dose calculation methods in ocular plaque brachytherapy,^13^ this study assumed that the spherically shaped eye consisting of water^14^ is located in a homogenous water medium. Eye anatomy and coordinates for critical structures in the eye were based on the COMS protocol (Fig. 2).^15^ The eye has an outer radius of 12 mm and the inner sclera surface has a radius of 11 mm. The macula (fovea) is located at 11 mm from the eye center in the inner sclera surface. The maximum separation between the macula and the center of the optic disc is 4 mm. Optic disc diameter in a fundus diagram is 1.5 mm.

### 2.C Parameters for calculations of optic disc dose

According to the COMS protocol, three parameters are required for calculations of optic disc dose.^15^ Basal dimension of tumor at center in the direction from optic disc (BD, parameter #1) and distance from optic disc to tumor margin (DT, parameter #2) are determined in a fundus diagram [Fig. 2(a)]. Apical height of the tumor determining a prescription depth (parameter #3) is usually measured using ultrasound [Fig. 2(b)].

### 2.D Calculations of optic disc dose for a prescription depth of 5 mm

In our previous study, using our in‐house brachytherapy dose calculation program, optic disc dose for seven (from 10 to 22 mm) standard COMS plaques loaded with ^125^I seeds (models: IsoAid Advantage IAI‐125A, Best Industries 2301 and Bebig I25.S16) was comprehensively investigated as a function of DT for various BDs.^16^ The in‐house program was developed using MATLAB software (vR2016a, MathWorks, Natick, MA) by incorporating the AAPM TG‐43U1 dosimetry formalism with a line source approximation in a homogeneous water medium^17^ and COMS seed coordinates from Table 1 in the AAPM TG‐129 report.^4^ The in‐house program was validated for benchmark calculations in the literature, demonstrating similar accuracy to three commercially available treatment planning systems which use the same dose calculation algorithm as our in‐house program.^16^ Then optic disc dose calculations were performed for a prescribed dose of 85 Gy normalized to a central‐axis depth of 5 mm for an irradiation time of 168 h.^16^

In this study, optic disc dose for notched COMS plaques loaded with ^125^I seeds was calculated. Of commercially available ^125^I seeds, the seed model used in our institution (IsoAid Advantage IAI‐125A, IsoAid, LLC, Port Richey, FL) was selected for this study. Using the validated in‐house program, dose calculations were performed in the same way as for standard COMS plaques for the following two cases: ***Case #1)*** six (from 12 to 22 mm) notched COMS plaques with one seed removed from standard COMS plaques and ***Case #2)*** five (from 14 to 22 mm) notched COMS plaques with two seeds removed from standard COMS plaques. As mentioned in the Section 2.A, the 10 mm COMS plaque was not included in Cases #1 and #2, and the 12 mm COMS plaque was not included in Case #2. For each notched COMS plaque, seed position number(s) removed from the corresponding standard COMS plaque are shown in Table 1. Once seed(s) are removed from standard COMS plaques, the prescription depth is not covered by the prescribed dose of 85 Gy. After seed(s) were removed, therefore, for the same irradiation time of 168 h, air kerma strength (S_k_) per seed needed to be increased (i.e., re‐normalized 85 Gy to the prescription depth of 5 mm) to ensure full coverage of the prescription depth in both cases.

### 2.E Generation of dose conversion factors for different prescription depths

A prescription depth is not always 5 mm and it is determined based on the tumor apex. According to the COMS protocol,^14^ a prescription depth is 5 mm for the tumor apex <5 mm and the apex for the tumor apex ≥5 mm. In the recent American Brachytherapy Society consensus guidelines,^18^ a prescription depth is the tumor apex for all medium‐sized choroidal melanomas. Considering a prescription depth ranging from 1 to 10 mm, dose conversion factors to estimate optic disc dose for different prescription depths are necessary.

First, dose conversion factors for different prescription depths were generated for standard COMS plaques. Using the in‐house program, optic disc dose calculations for standard COMS plaques were performed for various prescription depths from 1 to 10 mm in 1 mm intervals. Then ratios of total reference air kerma (TRAK) per seed to obtain 85 Gy for an irradiation time of 168 h to each prescription depth to that to 5 mm were taken as dose conversion factors.^16^ TRAK per seed is defined as the product of S_k_ per seed (U) and irradiation time (hours), and its unit is µGym^2^ (=U × hours).

Second, dose conversion factors for different prescription depths were generated for notched COMS plaques (Cases #1 and #2). Using the in‐house program, optic disc dose for notched COMS plaques was calculated for prescription depths from 1 to 10 mm in 1 mm intervals. Then dose conversion factors were obtained in the same way as for standard COMS plaques. After seed removal, the prescribed dose (85 Gy) and irradiation time (168 h) were kept the same as for the prescription depth of 5 mm.

### 2.F Calculations of optic disc dose reduction by notched COMS plaques

Optic disc dose reduction by notched COMS plaques (Cases #1 and #2) was calculated from optic disc doses for standard and notched COMS plaques for all prescription depths from 1 to 10 mm. Absolute optic disc dose reduction, $D_{\text{reduction}\;}^{\text{Abs}}\;\text{(Gy)}$, was computed for each case using Eq. (1):

(1) $$D_{\text{reduction}}^{\text{Abs}}{\;\text{(Gy) = D}}_{\text{standard}}-D_{\text{notch}}$$

where D_standard_ is optic disc dose for standard COMS plaques and D_notch_ is corresponding optic disc dose for notched COMS plaques. Relative optic disc dose reduction, $D_{\text{reduction}}^{\text{Rel}}\left(\text{\textbackslash{}\%}\;\right)$, was computed for each case using Eq. (2):

(2) $$D_{\text{reduction}}^{\text{Rel}}\left(\%\right)=\frac{D_{\text{standard}}-D_{\text{notch}}}{D_{\text{standard}}}\times 100$$

where D_standard_ and D_notch_ are the same as for the absolute dose reduction.

## 3 RESULTS

### 3.A Optic disc dose reduction for a prescription depth of 5 mm

#### 3.A.1 Case #1: one seed removal

**Fig. 3:**
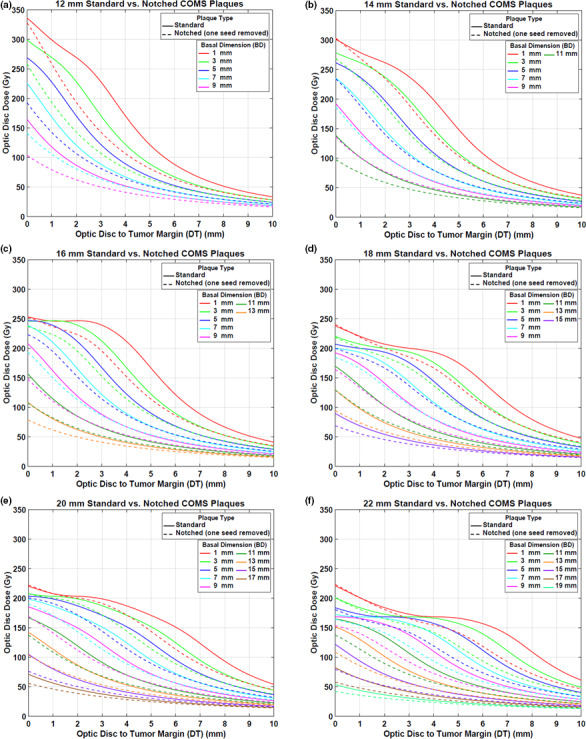
(a)–(f) Comparison of optic disc doses (Gy) between six standard Collaborative Ocular Melanoma Study (COMS) plaques and corresponding notched COMS plaques with one seed removed (Case #1). ^125^I (model IAI‐125A) seeds were loaded. The prescribed dose of 85 Gy for an irradiation time of 168 h was normalized at a depth of 5 mm.

Figures 3(a)–3(f) presents optic disc dose (dashed lines) as a function of DT for various BDs for six notched COMS plaques with one seed removed in comparison to that (solid lines) for corresponding standard COMS plaques. A prescription depth is 5 mm. As expected, when one seed is removed, optic disc dose is reduced.

**Fig. 4:**
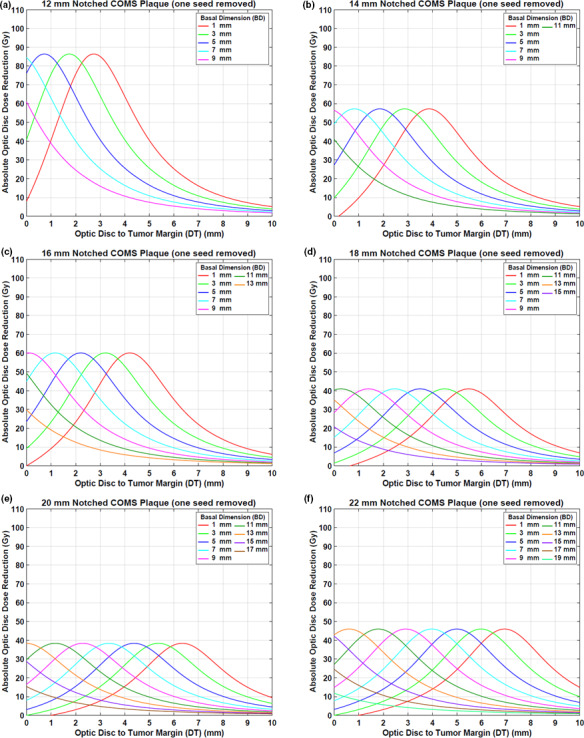
(a)–(f) Absolute optic disc dose reduction (Gy) by ^125^I (model IAI‐125A) notched Collaborative Ocular Melanoma Study plaques with one seed removed (Case #1). The prescribed dose of 85 Gy was normalized at 5 mm.

$D_{\text{reduction}}^{\text{Abs}}\;\text{(Gy)}\;$ by notched COMS plaques with one seed removed is shown in Figs. 4(a)–4(f). A prescription depth is 5 mm. The following unique trends for $D_{\text{reduction}}^{\text{Abs}}\;\text{(Gy)}\;$ are observed.

1. $D_{\text{reduction}}^{\text{Abs}}\;\text{(Gy)}\;$ increases with DT, reaches the maximum value (${\text{MaxD}}_{\text{reduction}}^{\text{Abs}}$) and then decreases with DT. For the largest 2‐3 BDs, however, dose reduction continuously decreases with DT. Examples include those for BDs of 7 and 9 mm in the 12 mm notched plaque [Fig. 4(a)] and those for BDs of 15, 17, and 19 mm in the 22 mm notched plaque [Fig. 4(f)].
2. Maximum absolute optic disc dose reduction (${\text{MaxD}}_{\text{reduction}}^{\text{Abs}}$) is usually larger with smaller plaques but does not continuously decrease with plaque size.
3. The magnitude of ${\text{MaxD}}_{\text{reduction}}^{\text{Abs}}$ does not vary with BD in each plaque except for those for the largest 2–3 BDs.
4. DT at which ${\text{MaxD}}_{\text{reduction}}^{\text{Abs}}$ occurs (DT_maxD_) decreases by about 1 mm with increasing BD by 2 mm. For example, for the 12 mm notched plaque, DT_maxD_ decreases from 2.7 to 1.7 mm as BD increases from 1 to 3 mm [Fig. 4(a)]. On the other hand, for the largest 2‐3 BDs, DT_maxD_ is always 0 mm or close to 0 mm. For instance, in the 12 mm notched plaque, DT_maxD_ becomes 0 mm when BD is 7 or 9 mm [Fig. 4(a)].
5. DT_maxD_ increases with plaque size for the same BD. For BD of 5 mm (blue lines in Fig. 4), DT_maxD_ are 0.7, 1.9, 2.2, 3.5, 4.4, and 5.0 mm for 12, 14, 16, 18, 20, and 22 mm plaques, respectively.

**Fig. 5:**
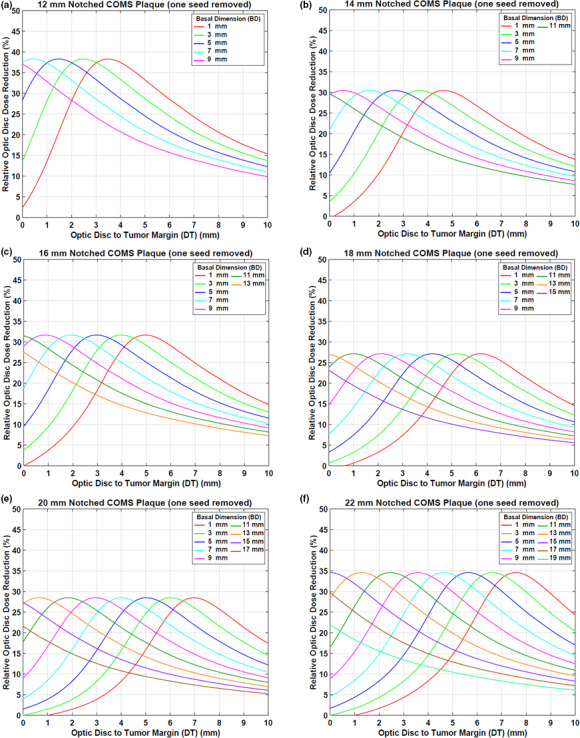
(a)–(f) Relative optic disc dose reduction (%) by ^125^I (model IAI‐125A) notched Collaborative Ocular Melanoma Study plaques with one seed removed (Case #1).

Corresponding $D_{\text{reduction}}^{\text{Rel}}\left(\text{\textbackslash{}\%}\;\right)$ is shown in Figs. 5(a)–5(f). ${\text{MaxD}}_{\text{reduction}}^{\text{Abs}}\left(\text{Gy}\right)$ (${\text{MaxD}}_{\text{reduction}}^{\text{Rel}}\left(\text{\textbackslash{}\%}\;\right))$ are 86.3 Gy (38.2%), 57.1 Gy (30.4%), 59.9 Gy (31.5%), 40.9 Gy (27.1%), 38.3 Gy (28.4%), and 45.5 Gy (34.3%) for 12, 14, 16, 18, 20, and 22 mm plaques, respectively, excluding those for the largest 2–3 BDs (Figs. 4 and 5).

#### 3.A.2 Case #2: two seeds removal

**Fig. 6:**
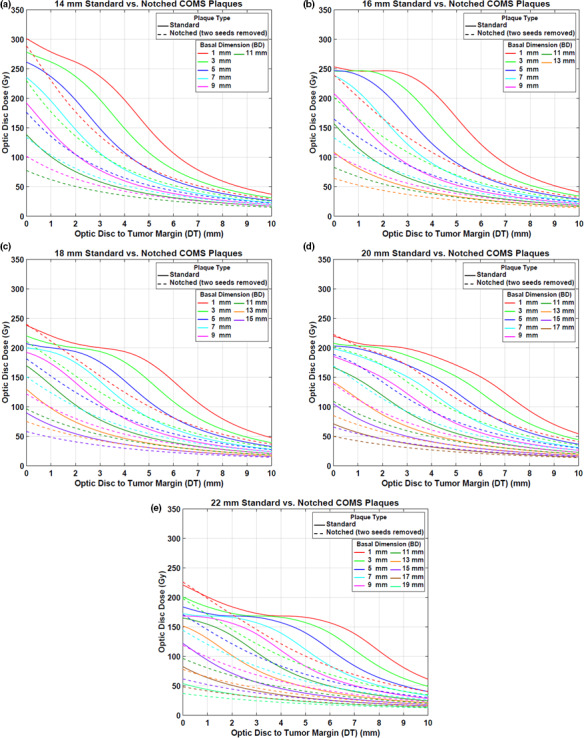
(a)–(e) Comparison of optic disc doses (Gy) between five standard Collaborative Ocular Melanoma Study (COMS) plaques and corresponding notched COMS plaques with two seeds removed (Case #2). ^125^I (model IAI‐125A) seeds were loaded. The prescribed dose of 85 Gy for an irradiation time of 168 h was normalized at 5 mm.

Figures 6(a)–6(e) displays optic disc dose (dashed lines) as a function of DT for various BDs for five notched COMS plaques with two seeds removed in comparison to that (solid lines) for corresponding standard COMS plaques. A prescription depth is 5 mm.

**Fig. 7:**
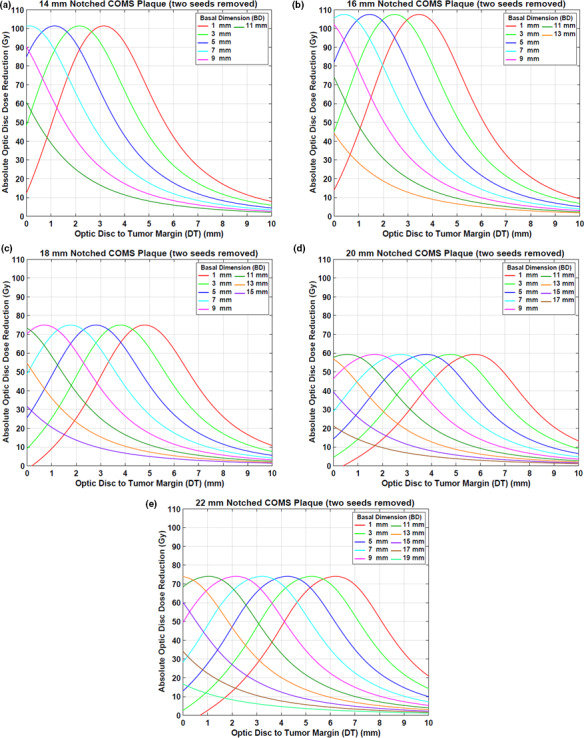
(a)–(e) Absolute optic disc dose reduction (Gy) by ^125^I (model IAI‐125A) notched Collaborative Ocular Melanoma Study plaques with two seeds removed (Case #2). The prescribed dose of 85 Gy was normalized at 5 mm.

$D_{\text{reduction}}^{\text{Abs}}\;\text{(Gy)}\;$ by notched COMS plaques with two seeds removed is shown in Figs. 7(a)–7(e). A prescription depth is 5 mm. The amount of dose reduction is larger but trends for dose reduction are similar to those in Case #1.

**Fig. 8:**
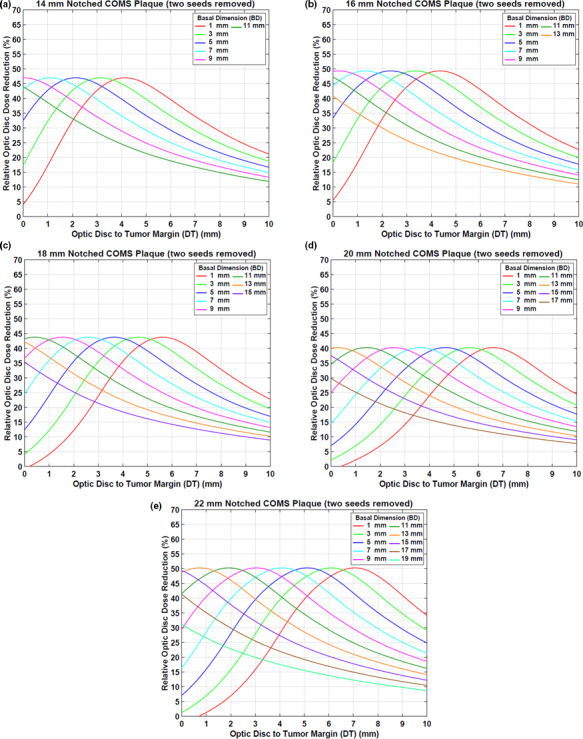
(a)–(e) Relative optic disc dose reduction (%) by ^125^I (model IAI‐125A) notched Collaborative Ocular Melanoma Study plaques with two seeds removed (Case #2).

Corresponding $D_{\text{reduction}}^{\text{Rel}}\left(\text{\textbackslash{}\%}\;\right)$ is shown in Figs. 8(a)–8(e). ${\text{MaxD}}_{\text{reduction}}^{\text{Abs}}\;\text{(Gy)}$ (${\text{MaxD}}_{\text{reduction}}^{\text{Rel}}\;\text{(\textbackslash{}\% )})$ are 101.4 Gy (47.0%), 107.5 Gy (49.3%), 75.0 Gy (43.7%), 59.4 Gy (40.2%), and 74.0 Gy (50.3%) for 14, 16, 18, 20, and 22 mm plaques, respectively, excluding those for the largest 2–3 BDs (Figs. 7 and 8).

### 3.B Dose conversion factors for different prescription depths

Dose conversion factors are presented in Table 2 for six standard COMS plaques, six notched COMS plaques with one seed removed (Case #1) and five notched COMS plaques with two seeds removed (Case #2). For plaques from 14 to 22 mm, dose conversion factors for Case #1 are similar to those for Case #2 within 2.4%. Compared with dose conversion factors for standard COMS plaques, those for notched COMS plaques from 14 to 22 mm are slightly higher (up to 2.7% for Case #1 and 3.4% for Case #2) at shallow (<5 mm) prescription depths and slightly lower (up to 1.1% for Case #1 and 1.4% for Case #2) at deeper (>5 mm) depths. However, trends for dose conversion factors are similar between standard and notched COMS plaques. The factors increase with increasing prescription depth. Also, the factors increase with increasing plaque size for a depth <5 mm, but decrease with increasing plaque size for depth >5 mm.

**Table 2:** Dose conversion factors (ratios of total reference air kerma per seed) for various prescription depths (1–10 mm in 1 mm intervals) for ^125^I (model IAI‐125A) standard Collaborative Ocular Melanoma Study (COMS) plaques, notched COMS plaques with one seed removed (Case #1) and notched COMS plaques with two seeds removed (Case #2). The data were normalized to those for a prescription depth of 5 mm

Prescription depth (mm)	Plaque size in diameter (mm)					
	12	14	16	18	20	22
Standard COMS plaques						
1	0.33	0.37	0.42	0.44	0.47	0.47
2	0.45	0.48	0.52	0.55	0.58	0.59
3	0.60	0.63	0.66	0.68	0.70	0.71
4	0.78	0.80	0.81	0.83	0.84	0.85
5	1.00	1.00	1.00	1.00	1.00	1.00
6	1.25	1.23	1.22	1.20	1.18	1.18
7	1.54	1.50	1.47	1.43	1.40	1.38
8	1.88	1.82	1.76	1.69	1.64	1.61
9	2.27	2.18	2.10	2.00	1.92	1.87
10	2.71	2.59	2.48	2.34	2.24	2.17
Notched COMS plaques with one seed removed (Case #1)						
1	0.33	0.36	0.41	0.44	0.47	0.47
2	0.45	0.48	0.52	0.55	0.57	0.58
3	0.60	0.62	0.65	0.68	0.70	0.70
4	0.78	0.80	0.81	0.83	0.84	0.84
5	1.00	1.00	1.00	1.00	1.00	1.00
6	1.26	1.24	1.22	1.20	1.19	1.18
7	1.55	1.51	1.48	1.43	1.40	1.38
8	1.89	1.83	1.77	1.70	1.65	1.62
9	2.28	2.20	2.11	2.01	1.94	1.89
10	2.73	2.61	2.50	2.36	2.26	2.19
Notched COMS plaques with two seeds removed (Case #2)						
1	Unavailable	0.36	0.41	0.43	0.46	0.46
2	Unavailable	0.48	0.52	0.55	0.57	0.57
3	Unavailable	0.62	0.65	0.67	0.69	0.70
4	Unavailable	0.80	0.81	0.83	0.84	0.84
5	Unavailable	1.00	1.00	1.00	1.00	1.00
6	Unavailable	1.24	1.22	1.20	1.19	1.18
7	Unavailable	1.51	1.47	1.43	1.40	1.39
8	Unavailable	1.83	1.77	1.70	1.65	1.62
9	Unavailable	2.19	2.11	2.01	1.94	1.89
10	Unavailable	2.60	2.49	2.36	2.26	2.20

### 3.C Optic disc dose reduction for different prescription depths

Optic disc dose reduction by notched COMS plaques increases with increasing prescription depth but its trends for the other prescription depths (1–4 mm & 6–10 mm) are similar to the five trends mentioned above for the depth of 5 mm (data not shown here). ${\text{MaxD}}_{\text{reduction}}^{\text{Abs}}$ increases with increasing prescription depth for each plaque in both cases (data not shown here).

### 3.D Options to reduce optic disc dose: clinical application of this study

A clinical example (BD = 3 mm, DT = 3 mm, and apical height = 3 mm) is given and a practical application of the results (Figures and Tables) obtained in this study for this example is demonstrated in Table 3. A prescribed dose is 85 Gy and a 14 mm COMS plaque loaded with ^125^I seeds (model IAI‐125A) is selected. Depending on the type of COMS plaque [standard plaque or notched plaque (Cases #1 or #2)] and prescription depth (3 mm or 5 mm), six clinical scenarios are possible and optic disc dose for each scenario can be estimated (Table 3). Of these, Scenario #1 (standard COMS plaque and prescription depth of 5 mm) and Scenario #6 (notched COMS plaque with two seeds removed and prescription depth of 3 mm) give the highest (197.6 Gy) and the lowest (65.0 Gy) optic disc doses, respectively. The optic disc dose difference between these two scenarios is 132.6 Gy. When 85 Gy is prescribed at 5 mm, dose reduction from optic disc dose (197.6 Gy) for the standard plaque is 56.9 Gy for one seed removal (Scenario #2) and 92.7 Gy for two seeds removal (Scenario #3). At a depth of 3 mm, dose reduction from optic disc dose (124.5 Gy) for the standard plaque is 37.3 Gy for one seed removal (Scenario #5) and 59.5 Gy for two seeds removal (Scenario #6). Although absolute dose reduction is larger for a depth of 5 mm, relative dose reduction is similar between the two depths [i.e., approximately 29% for one seed removal (Scenarios #2 and #5) and 47% for two seeds removal (Scenarios #3 and #6)].

**Table 3:** Six possible scenarios for a clinical example [BD = 3 mm, DT = 3 mm, and apical height = 3 mm in 14 mm Collaborative Ocular Melanoma Study (COMS) plaque] and corresponding estimated optic disc doses

Scenario #	Type of COMS plaque	Prescription depth (mm)	Dose conversion factor relative to depth of 5 mm	Estimated optic disc dose (Gy)	Absolute dose reduction from standard COMS plaque (Gy)	Relative dose reduction from standard COMS plaque (%)	Reference
1	Standard	5	1.00	197.6	Unavailable	Unavailable	Fig. 3(b)
2	Notched (one seed removed)	5	1.00	140.7	56.9	28.8	Fig. 3(b)
3	Notched (two seeds removed)	5	1.00	104.9	92.7	46.9	Fig. 6(a)
4	Standard	3	0.63	124.5	Unavailable	Unavailable	Fig. 3(b) and Table 2
5	Notched (one seed removed)	3	0.62	87.2	37.3	30.0	Fig. 3(b) and Table 2
6	Notched (two seeds removed)	3	0.62	65.0	59.5	47.8	Fig. 6(a) and Table 2

## 4 DISCUSSION

This study demonstrated that optic disc dose reduction by notched COMS plaques has its own trends (Figs. 4 and 7), and corresponding reasons for the five trends mentioned in the Results are as follows:

1. $D_{\text{reduction}}^{\text{Abs}}\;\text{(Gy)}\;$ is usually at maximum farther than 0 mm, whereas ${\text{MaxD}}_{\text{reduction}}^{\text{Abs}}$ occurs at 0 mm or close to 0 mm for the 2–3 largest BDs [Figs. 4 and 7]. This is attributed to unique patterns of optic disc dose curves as a function of DT, BD, and plaque size (Figs. 3 and 6). For small BDs in plaques ≥16 mm, there are regions in which dose does not change much with distance [Figs. 3(c)3, 6(f) and 3, 6(b)3, 6(e)], making ${\text{MaxD}}_{\text{reduction}}^{\text{Abs}}$ occur at farther distances than 0 mm (i.e., optimal DT_maxD_ exists for each plaque and for each BD). In contrast, for the largest 2–3 BDs, optic disc dose continuously decreases with DT for both standard and notched plaques (Figs. 3 and 6), thus, $D_{\text{reduction}}^{\text{Abs}}\;\text{(Gy)}\;$ continuously decreases with DT (Figs. 4 and 7) and ${\text{MaxD}}_{\text{reduction}}^{\text{Abs}}$ occurs at 0 mm or close to 0 mm. Figure 4 in Lee *et al*. explains this phenomenon with respect to an optic disc location relative to seed positions with varying DT.^16^
2. Since the total number of seeds do not continuously increase with plaque size, dose reduction does not continuously decrease with plaque size. Dose contribution per seed to a point of interest is higher for the 12 mm plaque (8 seeds in total) than for the 14 mm plaque (13 seeds in total) (TRAK per seed for a depth of 5 mm: 677.1 µGym^2^ vs. 438.1 µGym^2^) and thus, ${\text{MaxD}}_{\text{reduction}}^{\text{Abs}}$ by the 12 mm notched plaque is higher than that by the 14 mm notched plaque [Fig. 4(a) vs Fig. 4(b)]. On the other hand, ${\text{MaxD}}_{\text{reduction}}^{\text{Abs}}$ by the 14 mm plaque is similar to that by the 16 mm plaque (TRAK per seed for a depth of 5 mm: 438.1 µGym^2^ vs 459.8 µGym^2^) as both plaques contain the same total number of seeds (13 seeds in total) [Fig. 4(b) vs Fig. 4(c)]. Seed configurations which depend on the plaque size would also affect dose to a point of interest even though the effect would be small. The difference in dose reduction between 14 and 16 mm plaques is small for Case #1 [Fig. 4(b) vs Fig. 4(c)], whereas it is noticeable for Case #2 [Fig. 7(a) vs Fig. 7(b)].
3. ${\text{MaxD}}_{\text{reduction}}^{\text{Abs}}$ does not vary with BD except for those for the largest 2–3 BDs because the number of seeds and seed configurations do not change with BD for the same plaque size.
4. For small BDs, DT_maxD_ decreases with increasing BD because the dose‐invariant regions usually become shallower with increasing BD [Figs. 3(c)3, 6(f) and 3, 6(b)3, 6(e)]. The decrease in DT_maxD_ is almost constant (by 1 mm) with increasing BD by 2 mm because DT_maxD_ is only a function of distance from seeds to the optic disc for the same plaque size. For the largest 2‐3 BDs, as discussed in 1) above, ${\text{MaxD}}_{\text{reduction}}^{\text{Abs}}$ occurs at 0 mm or close to 0 mm and therefore, DT_maxD_ is 0 mm or close to 0 mm (Figs. 4 and 7).
5. DT_maxD_ increases with plaque size for the same BD because the distance from seed(s) to the optic disc increases with plaque size [Figs. 3(a)3, 6(f) and 3, 6(a)3, 6(e)].

**Fig. 9:**
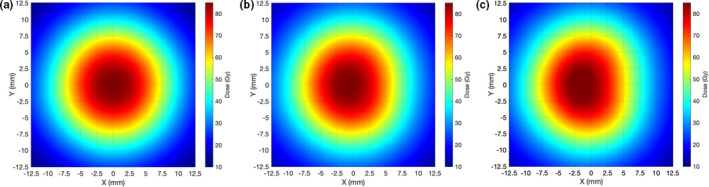
(a)–(c) Corresponding isodose clouds for the 14 mm Collaborative Ocular Melanoma Study plaques shown in Figs. 1(a)–1(c).

**Fig. 10:**
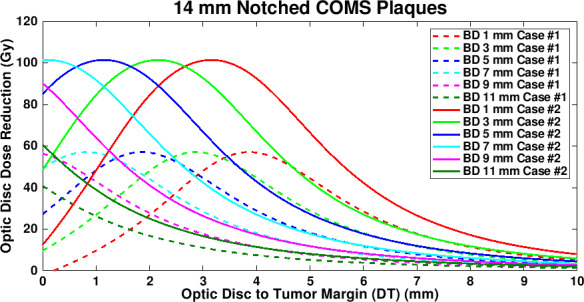
Comparison of optic disc dose reduction between Case #1 and Case #2 for 14 mm ^125^I (model IAI‐125A) notched Collaborative Ocular Melanoma Study plaques.

In this study, optic disc dose reduction for Case #1 and Case #2 was investigated. As more seeds near the optic disc are removed, dose contribution to the direction of the optic disc decreases. As a result, more optic disc dose reduction occurs for Case #2 (Fig. 9). However, differences in dose reduction between Case #1 and Case #2 do not continuously decrease with plaque size because the number of seeds and seed configurations depend on the plaque size. Due to more dose reduction to the optic disc in Case #2, DT_maxD_ becomes shallower (i.e., DT_maxD_ moves toward 0 mm) in Case #2 than in Case #1 for the same BD. However, the difference in DT_maxD_ between the two cases for the same BD is consistent (0.7–0.8 mm for 14, 16, 18, and 22 mm plaques and 0.5–0.6 mm for 20 mm plaque) because in each plaque, seed configurations are the same and the only difference is dose contribution per seed to the optic disc. Figure 10 shows the comparison of optic disc dose reduction between Case #1 and Case #2 for 14 mm COMS plaques. DT_maxD_ is shallower in Case #2 and its difference between the two cases is 0.7 mm. The 20 mm plaque has the largest number of seeds (24 seeds in total) and thus, the lowest TRAK per seed, resulting in the smallest differences in MaxD_reduction_ and in DT_maxD_ between the two cases. In both cases, after seed removal, the prescribed dose of 85 Gy was re‐normalized to each prescription depth but the irradiation time of 168 h was kept. As a result, S_k_ per seed was higher for notched COMS plaques than for corresponding standard COMS plaques and S_k_ per seed was higher for Case #2 than for Case #1 in the same COMS plaque.

${\text{MaxD}}_{\text{reduction}}^{\text{Abs}}$ has dependence on the prescription depth. ${\text{MaxD}}_{\text{reduction}}^{\text{Abs}}$ increases with increasing prescription depth because a deeper prescription depth requires higher TRAK per seed for both standard and notched COMS plaques and thus, dose reduction is larger at a deeper depth. From the results of this study, it is concluded that tumor size (i.e., BD and apical height (determining prescription depth)) has effects on optic disc dose reduction. For the same DT, as BD increases, $D_{\text{reduction}}^{\text{Abs}}\;\text{(Gy)}$ decreases (Figs. 4 and 7). As apical height increases, $D_{\text{reduction}}^{\text{Abs}}\;\text{(Gy)}$ increases (Table 2).

Juxtapapillary tumors as well as tumors close to the optic disc can benefit from notched COMS plaques in reducing dose to the optic disc. As shown in the Results, DT_maxD_ is 0 mm only for tumors with the largest 2–3 BDs and there exists optimal DT_maxD_ (nonzero mm) for tumors with small BDs. Therefore, notched COMS plaques would be the most beneficial to juxtapapillary tumors or circumpapillary tumors when their BDs are large and to peripapillary tumors (tumor <3.5 mm from the optic disc) or extrapapillary tumors (tumor ≥3.5 mm from the disc margin) when their BDs are small.^1^

The clinical example discussed in the Results showed that there are various scenarios in selecting COMS plaque type and prescription depth, and this example would help the clinician choose the best scenario to minimize radiation dose to the optic disc without treatment planning. As shown in Table 3, for tumors with apical height <5 mm, prescribing to the tumor apex gives lower optic disc dose than prescribing to a depth of 5 mm for both standard and notched COMS plaques. Depending on the prescription depth, however, a standard COMS plaque can reduce optic disc dose more than a notched COMS plaque. For example, Scenario #4 (standard COMS plaque and prescription depth of 3 mm) gives lower dose to the optic disc than Scenario #2 (notched COMS plaque and prescription depth of 5 mm) (124.5 vs 140.7 Gy). For tumors with apical height ≥5 mm, a prescription depth is always the tumor apex and notched COMS plaques would be always more beneficial.

## 5 CONCLUSION

This simulation study has comprehensively investigated dose reduction to the optic disc in ocular brachytherapy using ^125^I notched COMS plaques based on current clinical practice. Optic disc dose reduction by the use of notched COMS plaques has its own trends. The results (figures and tables) presented in this study would enable the clinicians (both ophthalmologist and radiation oncologist) to choose an adequate plaque type among standard ^125^I COMS plaques and notched ^125^I COMS plaques (with one seed removed and two seeds removed), and a prescription depth to minimize optic disc dose for a given clinical case.

## CONFLICT OF INTEREST

No conflict of interest.
